# Loneliness and sleep: A systematic review and meta-analysis

**DOI:** 10.1177/2055102920913235

**Published:** 2020-04-04

**Authors:** Sarah C Griffin, Allison B Williams, Scott G Ravyts, Samantha N Mladen, Bruce D Rybarczyk

**Affiliations:** Virginia Commonwealth University, USA

**Keywords:** health, insomnia, loneliness, mechanisms, sleep

## Abstract

Despite the mounting evidence linking loneliness with health, the mechanisms underlying this relationship remain obscure. This systematic review and meta-analysis on the association between loneliness and one potential mechanism—sleep—identified 27 relevant articles. Loneliness correlated with self-reported sleep disturbance (*r* = .28, 95% confidence interval (.24, .33)) but not duration, across a diverse set of samples and measures. There was no evidence supporting age or gender as moderators or suggesting publication bias. The longitudinal relationship between loneliness and sleep remains unclear. Loneliness is related to sleep disturbance, but research is necessary to determine directionality, examine the influence of other factors, and speak to causality.

In 2017, Dr. Vivek Murthy, 19th Surgeon General of the United States, named loneliness as the most common pathology he had encountered in his 3 years of service. The statement made headlines, but the awareness of social isolation as a health risk dates back decades. House et al. (1988) synthesized research at the time to argue that social relationships affect health and called for work on the social, psychological, and biological processes mediating this relationship. Subsequent research has bolstered their conclusion: multiple meta-analyses indicate that social factors predict morbidity and mortality (Holt-Lunstad et al., 2015; Sbarra et al., 2011; Shor et al., 2013).

However, there remains a dearth of research identifying mechanisms underlying the link between social relationships and health (Thoits, 2011). Loneliness, in particular, can be defined as *feeling* separate from others, and has been identified as one key aspect of social factors that influence health. Cacioppo and Hawkley (2003) proposed that sleep disturbance is a mechanism through which loneliness influences health, citing two studies led by Cacioppo (2002a, 2002b) in which lonely persons reported lower sleep quality and showed lower sleep efficiency and higher levels of wake time after sleep onset than non-lonely persons. Cacioppo and Hawkley (2003) argued that this sleep disturbance marked the loss of a fundamentally restorative behavior, thus affecting metabolic, neural, and hormonal processes. Evidence for this theory has subsequently been reviewed narratively (e.g. Cacioppo and Hawkley, 2003) but not systematically.

The present article aimed to provide a comprehensive review of the literature on the relationship between loneliness and sleep. A systematic review was conducted to describe the current literature on loneliness and sleep, with an emphasis on sampling and measurement. Meta-analytics were then used to quantitatively examine the cross-sectional literature on loneliness and sleep disturbance—defined as insomnia symptoms and subjective sleep quality—to generate mean effect sizes and assess for the presence of moderators (age and gender) and publication bias. This article represents a critical first step in synthesizing the current literature on the relationship between loneliness and sleep disturbance, thus seeking to address the 30-year-old question of the mechanisms that underly the connection between social relationships and health.

## Methods

This review was conducted according to the Preferred Reporting Items for Systematic reviews and Meta-Analyses (PRISMA) guidelines (Moher et al., 2009).

### Search strategy, eligibility criteria, and study selection

**Figure 1.**
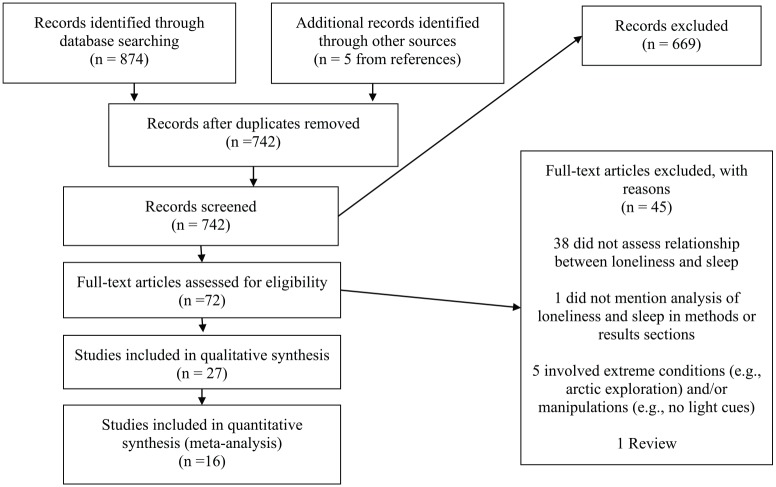
PRISMA flow diagram.

Searches were conducted in PubMed and PsycINFO between 2 and 7 February 2018. Reference sections of eligible studies were then reviewed (see Supplementary Appendix A for full searches). Eligibility criteria were (1) analysis of the relationship between sleep and loneliness (summarized in the results or tables), (2) quantitative methodology, (3) peer-reviewed, (4) adult sample, (5) written in English, (6) original research, (7) *not* sample with sleep apnea, and (8) *not* study with manipulation of temporal cues or other extreme conditions. Titles and abstracts, then full-text articles, were screened to determine eligibility. The search, screening, and selection process were conducted by the first author (S.G.; see Figure 1 for PRISMA flow diagram) then replicated by a co-author (S.R.). The replication process generated one additional article that was deemed ineligible because it pertained to cancer-related loneliness rather than loneliness more broadly (Adams et al., 2018).

### Data collection process

The following information was extracted from all studies: study design, sample characteristics (mean age, age range, gender, race/ethnicity, nationality), sampling method, measurement of sleep and loneliness, adjustments to model, findings on the relationship between sleep and loneliness, and effect sizes. The following information was extracted for longitudinal studies: length of follow-up, time points, attrition rate, and handling of attrition bias. This process was conducted by the first author (S.G.) then replicated by a co-author (A.B.W.). Bias was assessed qualitatively due to evidence that current tools fail to discriminate between methodological issues and deficient reporting (Shamliyan et al., 2010).

### Meta-analytic method

The principal summary measure was the correlation coefficient (*r*) between loneliness and sleep. Other summary measures were transformed to *r* or generated where possible (bivariate *β* = *r*; conversion using online resources from DeCoster, 2012 or Lipsey and Wilson, 2001). Confidence intervals (CIs) were estimated (Lipsey and Wilson, 2001) when not provided. Meta-analyses were conducted analyses in R x64 3.5.1 (following guidance from Quintana and Tanner-Smith, 2015; packages from Fisher and Tipton, 2015; Viechtbauer, 2010). Estimates were generated for sleep disturbance (defined as self-reported sleep quality and insomnia symptoms), self-reported sleep quality, and insomnia symptoms. Sleep quality was coded so that higher values represented greater problems with sleep quality. Sensitivity analyses were conducted, excluding studies with effect size estimates not meeting the *r* normality assumption and not excluding outliers. Risk of bias was assessed by visual examination of a funnel plot, the rank correlation test, and Egger’s regression test. Study homogeneity was assessed using the *Q*-statistic (Lipsey and Wilson, 2001; Quintana and Tanner-Smith, 2015). Moderators included age and gender. There were too few studies on other sleep outcomes to make quantitative analysis informative; however, these studies were included in the systematic review and described qualitatively. Similarly, we did not synthesize summary statistics across the longitudinal relationship due to the size (*k* = 8) and heterogeneity of this literature. Input data file, R code, and output are available on the Open Science Framework (https://osf.io/s6kbt/?view_only=6de74e9fa6ed40cfa027004e2cb66bb9).

## Results

### Sample

The total estimated sample size at baseline was 45,177 (if multiple studies reported on the same sample, the larger sample size was used in this estimate). Several articles used data from a larger study (e.g. the Chicago Health, Aging, and Social Relations Study). There were three cases of sample overlap across articles (Cacioppo et al., 2002a, 2002b; Hom et al., 2017a, 2017b; McHugh et al., 2011; McHugh and Lawlor, 2013). Sample characteristics for each study are summarized in Table 1 (see Supplementary Appendix B for the race/ethnicity of US samples).

**Table 1.** Sample characteristics.

Author (year)	Population	Sample size (analytic)	Mean age	Age range	% Male	% Female	Country
Aanes et al. (2011)	Two cohorts residing in Hordaland County, Norway	7074	Not reported	Approximately 46–50 or 70–75 (born: 1925–1927, 1950–1951; data collection: 1997–2000)	Estimate: 48.2	Estimate 51.8	Norway
Cacioppo et al. (2002a)	College students	64 (54 with sleep data from lab visit; 37 with sleep data at home)	Not reported	Not reported	61.1 lab; 62.1 home	38.9 lab; 37.8 home	United States
Cacioppo et al. (2002b)—Study 1	College students	89	19.26	18–24	50.56	49.44	United States
Cacioppo et al. (2002b)—Study 2	Chicago condominium	25	65.00	53–78	24.00	76.00	United States
Cheng et al. (2015)	Older adults living in rural villages in Chizhou, China	730	69.07	60–86	44.52	55.48	China
Christiansen et al. (2016)	Older adults in Denmark	8593	73.00	65–103	49.00	51.00	Denmark
Chu et al. (2016)	College students	552 (538)	21.53	18–34	25.50	74.50	South Korea
Davis and Shuler (2000)	Homeless women	50	29.90	18–44	0.00	100.00	United States
Hawkley et al. (2010)	Residents of Cook County, Illinois (Chicago)	229 (215)	57.40	50–68^a^	47.60	52.40	United States
Hayley et al. (2017)	Higher education students in Norway	12,043	Not reported	18–34	33.50	66.50	Norway
Hays and DiMatteo (1987)	College students	199	21.00	17–48	38.20	61.80	United States
Hom et al. (2017a)—Study 1	Military services members and veterans	937	38.20	18–88	82.10	17.90	United States
Hom et al. (2017a)—Study 2	Army recruiters	3386	29.91	20–57	91.50	8.50	United States
Hom et al. (2017a)—Study 3	Military veterans	417	50.73	20–98	67.80	32.20	United States
Hom et al. (2017b)—Study 1	Undergraduate students	747 (666)	18.90	18–33	63.00	37.00	United States
Hom et al. (2017b)—Study 2	Army recruiters	2785	29.90	20–57	91.90	8.10	United States
Hom et al. (2017b)—Study 3	Adults with a history of suicidality/depression	208	19.38	18–36	19.70	80.30	United States
Hom et al. (2017b)—Study 4	Adult psychiatric outpatients	343	26.78	18–71	39.50	60.50	United States
Hom et al. (2017b)—Study 5	Young adults at elevated suicide risk	326	22.17	18–37	82.20	17.80	United States
Hom et al. (2017b)—Study 6	College students	183^b^ (151)	19.00	17–29	45.90	54.10	United States
Jacobs et al. (2006)	West Jerusalem residents born between June 1920 and May 1921	452 (290)	70.00	Single cohort	51.72	48.28	Jerusalem
Jaremka et al. (2014)—Study 1	Cancer clinics at the Ohio State University—cancer patients and noncancer controls	115	56.77	30–88	17.00	83.00	United States
Jaremka et al. (2014)—Study 2	(1) Older adults caring for a spouse with Alzheimer’s disease or related dementia; (2) non-caregiver controls	229	69.68	35–91	28.00	72.00	United States
Kurina et al. (2011)	Hutterite adults living on two colonies in South Dakota	130 (95)	39.80	19–84	45.00	55.00	United States
Matthews et al. (2017)	Birth Cohort of British Twins	2232	18.40	Not applicable (single cohort)	Not reported but appears to be approximately even		United Kingdom
McHugh et al. (2011)	Irish community-dwelling adults over 60	505	73.33	Not reported (over age 60)	31.70	68.30	United Kingdom
McHugh and Lawlor (2013)	Irish community-dwelling adults over 60	624 (447)	73.32	Not reported	31.00	69.00	United Kingdom
O’Connell (2016)	Online—Irish, American, European, Canadian, Australian	118	30.60	18–59	32.20	67.80	92.4% Irish
Segrin and Passalacqua (2010)	College students, acquaintances of college students, parents of high school athletes	265	41.45	19–85	47.55	52.45	United States
Segrin and Domschke (2011)	College students, acquaintances of college students	224	41.22	18–81	34.82	65.18	United States^c^
Segrin and Burke (2015)	College students	510	45.55	Not reported	50.00	50.00	United States
Smith et al. (2010)	University community	97	21.6	Not reported	28.87	71.13	Australia
Steptoe et al. (2004)	London-based civil servants aged 35–55 in 1985–1988	240	Not reported	47–59	53.75	46.25	United Kingdom
Stickley et al. (2015)	Moscow residents	1190	Not reported	Not reported (over age 18)	42.86	57.14	Russia
Yu et al. (2017)	Taiwanese adults aged 60 and older	1023 (639)	66.14	54–80	57.67	42.33	Taiwan
Zawadzki et al. (2013)—Study 3	College students	218	20.30	Not reported	24.31	75.69	United States
Zawadzki et al. (2013)—Study 4	College students	360 (334)	21.20	Not reported	22.75	77.25	United States

Overlapping samples are highlighted the same shade of gray.

Sample size estimates are at baseline for longitudinal studies.

Mean ages and age ranges are at baseline.

See Supplementary Appendix H for full citations of articles.

a From Hawkley et al. (2008).

b Sample with data at both time points; list-wise deletion used for longitudinal analyses (yielding sample size of 151), but not specified if cross-sectional analyses used the larger dataset.

c Not reported but authors are from the United States.

### Measurement

Studies examined different dimensions of sleep, to include sleep duration, sleep quality, insomnia symptoms, sleep satisfaction, and sleep adequacy; two studies measured sleep quality objectively (Cacioppo et al., 2002a; Kurina et al., 2011). Loneliness was measured both directly (i.e. using the term “lonely”) and indirectly (avoiding the term “lonely” due to potential stigma). See Supplementary Appendix C for a summary of the measures for each study with a brief note on quality; see Supplementary Appendix D for psychometric properties of scales.

### Cross-sectional relationship between loneliness and sleep

**Figure 2.**
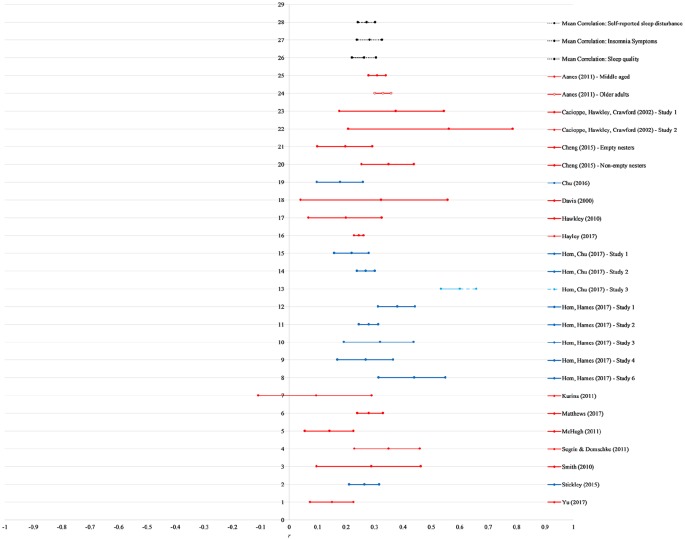
Forest plot of the effect sizes (self-reported sleep quality and insomnia symptoms) of studies included in the meta-analysis. Shading signifies the type of outcome, in order from top: mean effect sizes (black, dotted line), sleep quality (red), insomnia symptoms (blue). The third study presented in Hom, Chu et al. paper (light blue) is an outlier and was excluded from main analyses.

The random-effects model of correlation between loneliness and sleep disturbance (where sleep disturbance is defined as impaired sleep quality and insomnia symptoms, number of studies (*k*) = 24, and number of participants (*n*) = 34,254) showed a medium-sized association, *r* = .27, 95% CI (.24, .30). The random-effects model of the correlation between loneliness and subjective sleep quality (*k* = 15, *n* = 24,018, *r* = .26, 95% CI (.22, .31)) and insomnia symptoms (*k* = 9, *n* = 10,236, *r* = .28, 95% CI (.24, .33)) similarly showed medium-sized associations. There was evidence of heterogeneity in effect size across all estimates (*Q*-statistics at *p* < .05). See Supplementary Appendix E for details on these analyses and see Figure 2 for forest plot.

**Figure 3.**
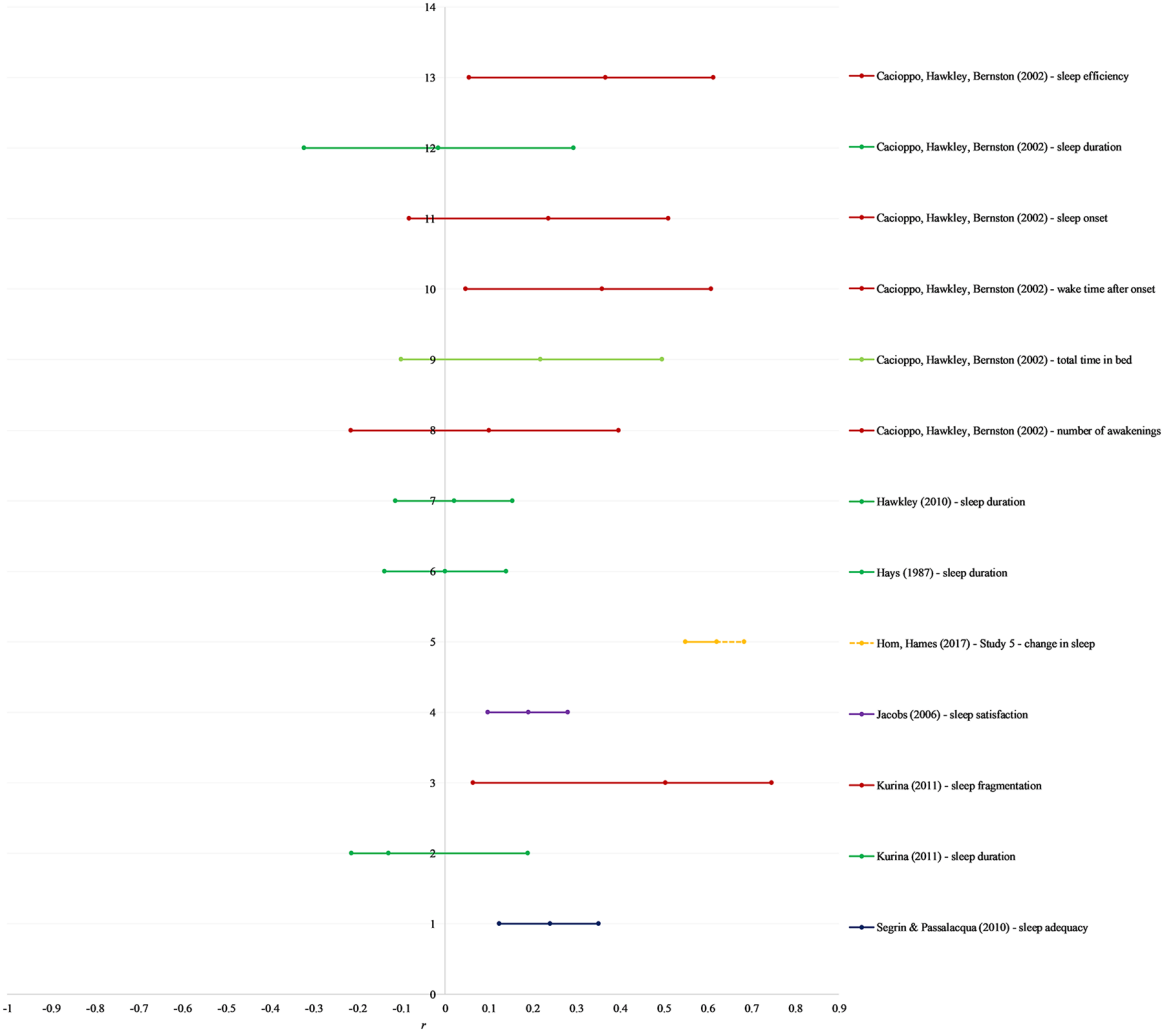
Forest plot of additional sleep outcome effect sizes. Each sleep outcome is labeled in the key. Shading signifies type of sleep outcome, in order from top: objective sleep quality (red), sleep duration (medium green), time in bed (light green), change in sleep (yellow), sleep satisfaction (purple), sleep adequacy (navy blue).

All but one study (Kurina et al., 2011) found a bivariate correlation between loneliness and subjectively measured sleep quality and insomnia symptoms. Both studies that measured sleep quality objectively (Cacioppo et al., 2002a: polysomnography; Kurina et al., 2011: actigraphy) found a bivariate association for a dimension of sleep quality. All studies that assessed sleep adequacy (Jaremka et al., 2014; Segrin and Passalacqua, 2010), sleep satisfaction (Jacobs et al., 2006), or change in sleep (Hom et al., 2017b) detected a bivariate association between these variables and loneliness. None of the studies that examined sleep duration detected an effect (Cacioppo et al., 2002a; Hawkley et al., 2010; Hays and DiMatteo, 1987; Kurina et al., 2011). However, Christiansen et al. (2016) found that sleep duration mediated the relationship between loneliness and both diabetes and cardiovascular disease. See Figure 3 for a forest plot of effect sizes not included in analyses.

#### Adjustments

Most studies did not control for potential confounds. See Table 2 for a narrative summary of findings on the relationship between loneliness and sleep with adjustments for confounds. Adjustments varied substantially across studies. A common adjustment was for depression, which attenuated the relationship between loneliness and both sleep quality and insomnia symptoms across all studies accounting for this factor.

**Table 2.** Narrative summary of results of studies that accounted for other factors.

Author (year)	Narrative summary of result
Cheng et al. (2015)	No significant association between sleep quality and loneliness when controlling for age, gender, education, occupation, income, marital status, depression, social support, and quality of life.
Hayley et al. (2017)	Association attenuated when controlling for age, gender, income, physical exercise, smoking, BMI, alcohol use, program, semester, social factors, anxiety, and depression.
Hom et al. (2017b)—Study 1	No significant association between insomnia and loneliness when controlling for depression.
Hom et al. (2017b)—Study 2	Association between insomnia and loneliness attenuated but still significant when controlling for perceived burdensomeness.
Hom et al. (2017b)—Study 3	No significant association between insomnia and loneliness when controlling for depression.
Hom et al. (2017b)—Study 4	No significant association between insomnia and loneliness when controlling for depression.
Kurina et al. (2011)	Association between sleep fragmentation and loneliness attenuated when controlling for age, sex, BMI, risk of sleep apnea, and negative effect.
Matthews et al. (2017)	Association between sleep quality and loneliness attenuated when controlling for social isolation, depression, anxiety, alcohol use, ADHD, PTSD, not being in employment, education, or training, and being the parent of an infant.
McHugh et al. (2011)	Loneliness not a significant predictor of poor versus good sleep quality when controlling for neuroticism, anxiety, depression, stress, age, polypharmacy, pain, gender, and age-adjusted comorbidity.
Segrin and Burke (2015)	Significant association between sleep quality and loneliness when controlling for depression (bivariate relationship not reported).
Smith et al. (2010)	No significant association between sleep quality and loneliness over and above depression, anxiety, and stress.
Steptoe et al. (2004)	Significant association between sleep quality and loneliness when controlling for age, sex, marital status, and grade of employment (bivariate relationship not reported).
Stickley et al. (2015)	Association between insomnia and loneliness attenuated when controlling for sex, age, marital status, education, household size, economic situation, social contacts, association membership, and social support.
Yu et al. (2017)	No significant difference on adjusted sleep quality score (age, sex, education, smoking, alcohol use, exercise, blood pressure, heart disease, stroke, ADLs/IADLs, cognitive impairment, depressive symptoms) in persons with high versus low loneliness.
Zawadzki et al. (2013)—Study 3	The direct path between loneliness and poor sleep quality was no longer significant when rumination and anxiety were included as mediators.

BMI: body mass index; ADHD: attention deficit/hyperactivity disorder; PTSD: post-traumatic stress disorder; ADLs: activities of daily living; IADLs: instrumental activities of daily living.

#### Outliers

The Baujat plot identified the third study by Hom et al. (2017a) as an outlier (see Supplementary Appendix F). The study was thus excluded from main analyses but included in sensitivity analyses.

#### Moderators

There was no evidence that mean age (*Q*(1) = 0.30, *p* = .58) or gender (*Q*(1) = 0.24, *p* = .63) moderated the association between loneliness and sleep disturbance.

#### Risk of bias across studies

The risk of publication bias was examined for the cross-sectional literature using a funnel plot, rank correlation test, and Egger’s regression test. Visual inspection of the funnel plot (see Supplementary Appendix G), the rank correlation test (Kendall’s tau = .10, *p* = .50), and the Egger’s regression test (*z* = 1.05, *p* = .29) did not suggest publication bias.

#### Sensitivity analyses

Excluding all estimates that did not meet the statistical assumptions of the *r* statistic did not appreciably change results. Including the Hom et al. (2017b) study that was previously deemed to be an outlier inflated effect size estimates. See Supplementary Appendix E for details.

### Longitudinal relationship between loneliness and sleep

Only eight studies evaluated loneliness as a risk factor for sleep difficulties. These studies varied considerably in terms of measures, sample, length of follow-up (3 months to 7 years), attrition rates (5.5%–56.1%), handling of potential confounds, and handling of attrition. Table 3 contains a narrative summary of the findings of each study, which varied. Two studies led by Hom et al. (2017b) examined the inverse relationship—sleep problems as a risk factor for loneliness. In the first study, change in sleep did not predict loneliness. In the second, insomnia symptoms predicted loneliness 5 weeks later, though not when controlling for depression.

**Table 3.** Narrative summary of longitudinal studies.

Author (year)	Narrative summary of findings	% Lost to follow-up	Handling of attrition
Hom et al. (2017b)—Study 5	Baseline loneliness did not significantly predict endorsement of a change in sleep at 1 month or 6 months when controlling for baseline endorsement of a change in sleep; endorsement of a change in sleep at baseline did not predict loneliness at 1 month or 6 months when controlling for baseline loneliness.	56.13	Not specified (data after 6 months not included).
Hom et al. (2017b)—Study 6	Baseline loneliness predicted insomnia 5 weeks later when controlling for baseline insomnia symptoms and anxiety; baseline insomnia predicted loneliness 5 weeks later when controlling for baseline loneliness and anxiety. However, neither loneliness nor insomnia predicted the other when controlling for baseline depression.	17.49^a^	Analyses conducted only with participants who completed both data points.
Jacobs et al. (2006)	Baseline loneliness predicted sleep satisfaction 7 years later when controlling for baseline sleep satisfaction, depression, self-rated health, economic problems, obesity, and back pain; baseline sleep satisfaction predicted loneliness 7 years later but not when controlling for depression, health, fatigue, medical conditions, sleeping medications, activity, and gender.	35.84	Not specified.
Jaremka et al. (2014)—Study 1	Loneliness did not predict change in sleep quality over 1 year.	13.91^b^	Not specified.
Jaremka et al. (2014)—Study 2	Loneliness predicted decline in sleep adequacy over time (3-year follow-up).	12.23^b^	Used analysis (GEE) that enabled the inclusion of participants with partially missing data.
McHugh and Lawlor (2013)	Baseline loneliness predicted sleep quality approximately 2 years later when controlling for sleep quality at baseline, age, gender, and comorbidities.	28.37	Applied an attrition weight to apply to longitudinal data.
Yu et al. (2017)	Baseline loneliness did not predict change in sleep quality over 6 years when controlling for age, sex, education, smoking, alcohol use, exercise, blood pressure, heart disease, stroke, baseline sleep quality, ADLs/IADLs, cognitive impairment, isolation, and depression.	37.54	Examined differences in those lost versus not lost to follow-up.
Zawadzki et al. (2013)—Study 4	Change in loneliness predicted change in anxiety, which in turn predicted change in sleep over 3 months.	5.56	Analyses conducted only with participants who completed both data points.

GEE: generalized estimating equation; ADLs: activities of daily living; IADLs: instrumental activities of daily living.

a Estimate—attrition rate not specified; calculation made using percentage of missing data at either baseline or follow-up.

b Estimate—attrition rate not specified; calculation made using the degrees of freedom for longitudinal analyses to estimate *n* at follow-up.

## Discussion

This review aimed to synthesize the research on the relationship between loneliness and sleep. Studies examined different sleep outcomes, to include quality, duration, insomnia symptoms, adequacy, satisfaction, and change in sleep. Loneliness correlated with higher self-reported sleep disturbance (defined as impaired sleep quality and insomnia symptoms). Loneliness was also associated with sleep inadequacy and dissatisfaction, but not sleep duration. However, there was no evidence that the relationship between loneliness and sleep disturbance was moderated by age or gender. There is, as of yet, insufficient evidence to identify loneliness as a risk factor for sleep difficulties, due to the inconsistency of results and methodology across this small (*k* = 8) set of studies.

### Sample

A strength of the current body of literature is the wide array of samples, from agrarian Anabaptists to higher education students in Norway. The diversity of samples suggests that the association between loneliness and sleep is robust, and likely not limited to specific populations. However, this requires empirical examination: particularly of import in the United States is whether research can speak to the US population. None of the samples were representative of the US population, necessitating research using a nationally representative sample.

### Measurement

A second strength of the cross-sectional body of literature is the use of both subjective and objective sleep measures. Subjective and objective measures tap into different parts of the sleep experience, and thus are both important to collect. Moreover, the use of different types of measures helps to mitigate the potential bias stemming from the limitations of each measure. Measures of loneliness varied across studies, ranging in quality from single-items to scales with strong psychometric properties. Scales also varied in whether they assessed loneliness directly or indirectly. Direct measures of loneliness use the term “lonely,” whereas indirect measures circumvent this word (e.g. “Do you feel alone?”). The advantage of direct measures of loneliness is that they tap directly into the construct of interest, whereas indirect measures are more likely to tap into related constructs, such as social support. However, responses on direct measures of loneliness could be biased by the stigmatization of loneliness (Shiovitz-Ezra and Ayalon, 2012). The use of both direct and indirect measures is thus a strength of the current body of literature: if findings replicate across both types of measure it is likely that the findings do not stem from the bias of either.

### Cross-sectional relationship between loneliness and sleep

This review found a medium-sized effect for the cross-sectional association between loneliness and sleep disturbance, both in terms of lower sleep quality and higher insomnia symptoms. Controlling for other factors, especially depression, attenuated the association between loneliness and sleep disturbance, indicating that the association is not independent of depression. However, simply controlling for depression does not speak to the interplay between depression, loneliness, and sleep disturbance: further research is necessary to examine how loneliness and sleep disturbance occur in the context of depression and other factors, such as age, race, and gender.

There was no evidence that either age or gender moderated the association between loneliness and sleep disturbance. The finding that gender does not moderate the connection between sleep and loneliness is consistent with previous meta-analyses examining psychosocial factors—to include social relationships, isolation, loneliness, and social support—and health, which consistently fail to identify gender as a moderator (Holt-Lunstad et al., 2010, 2015; Shor et al., 2013). However, this literature is mixed in terms of the identification of age as a moderator: a meta-analysis by Holt-Lunstad et al. (2015) showed that the link between mortality, loneliness, isolation, and living alone was stronger in *younger* samples; a meta-analysis by Shor et al. (2013) showed that the link between mortality and social support was stronger in *older* samples; and a meta-analysis by Holt-Lunstad et al. (2010) did not find differences in the strength of the connection between mortality and social relationships according to age. These apparent contradictions in the literature—and the fact that the present review did not detect age as moderator—likely stem from the constellation of risk and resilience factors in later adulthood. Older adulthood is marked both by increased strengths—via enhanced coping strategies—and increased vulnerabilities—via decreased ability to recover from the sustained arousal accompanying stressors (Charles, 2010). As such, it is possible that both protective and detrimental processes are occurring in older adulthood, which in turn shape the connection between loneliness and sleep disturbance in opposing directions.

### Longitudinal relationship between loneliness and sleep

Eight studies evaluated loneliness as a risk factor for sleep difficulties, with differing conclusions, as well as two studies that assessed the inverse relationship, sleep disturbance as a risk factor for loneliness. These studies differed in terms of their outcome of interest, length of follow-up, measures, samples, analyses, and handling of attrition and potential confounds. The variability in methodology, in tandem with the paucity of studies, makes it difficult to pinpoint which factors are driving the differences in results.

### Limitations

The present systematic review and meta-analysis must be interpreted in light of its limitations. First, the review does not include gray literature or unpublished findings. However, there was no evidence of publication bias (as examined by visual inspection of a funnel plot, the rank correlation test, and Egger’s regression test). Second, the review is limited in its ability to speak to the relationship between sleep disturbance and other aspects of social relationships, in particular isolation. The correlation between loneliness and isolation is modest yet their effects on mortality are comparable (Holt-Lunstad et al., 2015). As such, loneliness and isolation likely influence health through different, though potentially overlapping, mechanisms. This review speaks to one potential pathway through which isolation could affect health: isolation leads to loneliness, which in turn influences sleep disturbance; additional research is necessary to pinpoint other pathways.

Third, this review only examines two potential moderators: age and gender. However, there was evidence of study heterogeneity, suggesting the presence of moderators. Further research is necessary to identify and evaluate these moderators, which could include socioeconomic status, comorbid health conditions, depression, anxiety, and race. Fourth, this review speaks only to loneliness as a correlate and risk factor for sleep disturbance. However, these two lines of evidence are necessary but not sufficient to establish sleep disturbance as a mechanism through which loneliness deteriorates health. The present review was limited to observational literature; experimental research is necessary to speak to causality and therefore mechanisms.

## Conclusion

There is a medium-sized correlation between loneliness and sleep disturbance, but not sleep duration, across a wide array of measures and samples. Accounting for other factors—in particular depression—attenuated this association across all studies that assessed for potential confounds. The literature on loneliness as a risk factor for sleep disturbance is inconclusive due to variability in methodology and findings. This review indicates that loneliness is associated with impaired sleep quality and insomnia symptoms. Further research is necessary to determine directionality (i.e. whether loneliness precedes sleep disruption or the reverse (Simon and Walker, 2018)), assess how other factors such as depression play into this association, and speak to causality using experimental design. The inconclusiveness of the current literature precludes the ability to draw conclusions as to whether sleep disturbance is a mechanism for the connection between loneliness and health. However, this review represents a critical step in organizing and evaluating the current research with bearing on sleep disturbance as a mechanism, thus seeking to fill a research gap that has remained largely unaddressed for the past 30 years: how social relationships shape health.

## Supplemental Material

Supplementary_-_Appendix_A._Searches – Supplemental material for Loneliness and sleep: A systematic review and meta-analysis Click here for additional data file. Supplemental material, Supplementary_-_Appendix_A._Searches for Loneliness and sleep: A systematic review and meta-analysis by Sarah C Griffin, Allison B Williams, Scott G Ravyts, Samantha N Mladen and Bruce D Rybarczyk in Health Psychology Open

Supplementary_-_Appendix_B.__Ethnicity_for_studies_conducted_in_the_U.S. – Supplemental material for Loneliness and sleep: A systematic review and meta-analysis Click here for additional data file. Supplemental material, Supplementary_-_Appendix_B.__Ethnicity_for_studies_conducted_in_the_U.S. for Loneliness and sleep: A systematic review and meta-analysis by Sarah C Griffin, Allison B Williams, Scott G Ravyts, Samantha N Mladen and Bruce D Rybarczyk in Health Psychology Open

Supplementary_-_Appendix_C._Measures – Supplemental material for Loneliness and sleep: A systematic review and meta-analysis Click here for additional data file. Supplemental material, Supplementary_-_Appendix_C._Measures for Loneliness and sleep: A systematic review and meta-analysis by Sarah C Griffin, Allison B Williams, Scott G Ravyts, Samantha N Mladen and Bruce D Rybarczyk in Health Psychology Open

Supplementary_-_Appendix_D._Psychometric_properties_of_scales – Supplemental material for Loneliness and sleep: A systematic review and meta-analysis Click here for additional data file. Supplemental material, Supplementary_-_Appendix_D._Psychometric_properties_of_scales for Loneliness and sleep: A systematic review and meta-analysis by Sarah C Griffin, Allison B Williams, Scott G Ravyts, Samantha N Mladen and Bruce D Rybarczyk in Health Psychology Open

Supplementary_-_Appendix_E._Summary_of_meta-analyses – Supplemental material for Loneliness and sleep: A systematic review and meta-analysis Click here for additional data file. Supplemental material, Supplementary_-_Appendix_E._Summary_of_meta-analyses for Loneliness and sleep: A systematic review and meta-analysis by Sarah C Griffin, Allison B Williams, Scott G Ravyts, Samantha N Mladen and Bruce D Rybarczyk in Health Psychology Open

Supplementary_-_Appendix_F._Baujat – Supplemental material for Loneliness and sleep: A systematic review and meta-analysis Click here for additional data file. Supplemental material, Supplementary_-_Appendix_F._Baujat for Loneliness and sleep: A systematic review and meta-analysis by Sarah C Griffin, Allison B Williams, Scott G Ravyts, Samantha N Mladen and Bruce D Rybarczyk in Health Psychology Open

Supplementary_-_Appendix_G._Funnel_Plot – Supplemental material for Loneliness and sleep: A systematic review and meta-analysis Click here for additional data file. Supplemental material, Supplementary_-_Appendix_G._Funnel_Plot for Loneliness and sleep: A systematic review and meta-analysis by Sarah C Griffin, Allison B Williams, Scott G Ravyts, Samantha N Mladen and Bruce D Rybarczyk in Health Psychology Open

Supplementary_-_Appendix_H._Citations_for_Articles_Identified_in_Systematic_Review – Supplemental material for Loneliness and sleep: A systematic review and meta-analysis Click here for additional data file. Supplemental material, Supplementary_-_Appendix_H._Citations_for_Articles_Identified_in_Systematic_Review for Loneliness and sleep: A systematic review and meta-analysis by Sarah C Griffin, Allison B Williams, Scott G Ravyts, Samantha N Mladen and Bruce D Rybarczyk in Health Psychology Open
