# Supplementary material for: Loneliness and sleep: A systematic review and meta-analysis
Source: Health Psychol Open. 2020 Apr 4;7(1):2055102920913235. doi: 10.1177/2055102920913235 (PMC7139193; doi:10.1177/2055102920913235)
Supplement: Supplementary_-_Appendix_A._Searches – Supplemental material for Loneliness and sleep: A systematic review and meta-analysis [file Supplementary_-_Appendix_A._Searches.pdf]

Appendix A

Searches

Pubmed

(((((((((((((((((((sleep[MeSH Terms]) OR sleep[Title/Abstract])) AND ((((((((((Social Isolation[MeSH Terms]) OR isolation[Title/Abstract]) OR "social network"[Title/Abstract]) OR "network size"[Title/Abstract]) OR "social participation"[Title/Abstract]) OR loneliness[Title/Abstract]) OR "living alone"[Title/Abstract])))))))))) AND Humans[Mesh])) NOT (((((((Infant[MeSH Terms]) OR Child[MeSH Terms]) OR Adolescent[MeSH Terms])) OR pregnancy) OR sleep apnea) AND Humans[Mesh])) AND Humans[Mesh])) NOT (Review[Publication Type] AND Humans[Mesh])) AND Humans[Mesh] AND English[lang])) NOT Case Reports[Publication Type]) AND Humans[Mesh] AND English[lang]) Filters: Humans; English

- Search Date: 2.2.18; 491 results

((((((((((((((((((((((((insomnia[Title/Abstract])) AND ((((((((((Social Isolation[MeSH Terms]) OR isolation[Title/Abstract]) OR "social network"[Title/Abstract]) OR "network size"[Title/Abstract]) OR "social participation"[Title/Abstract]) OR loneliness[Title/Abstract]) OR "living alone"[Title/Abstract])))))))))) AND Humans[Mesh])) NOT (((((((Infant[MeSH Terms]) OR Child[MeSH Terms]) OR Adolescent[MeSH Terms])) OR pregnancy) OR sleep apnea) AND Humans[Mesh])) AND Humans[Mesh])) NOT (Review[Publication Type] AND Humans[Mesh])) AND Humans[Mesh] AND English[lang])) NOT Case Reports[Publication Type]) AND Humans[Mesh] AND English[lang])) AND Humans[Mesh] AND English[lang])) NOT Qualitative Research[MeSH Terms]) AND Humans[Mesh] AND English[lang])) NOT ((((((((((((((((((((((((sleep[MeSH Terms]) OR sleep[Title/Abstract])) AND ((((((((((Social

Isolation[MeSH Terms]) OR isolation[Title/Abstract]) OR "social network"[Title/Abstract]) OR "network size"[Title/Abstract]) OR "social participation"[Title/Abstract]) OR loneliness[Title/Abstract]) OR "living alone"[Title/Abstract])))))))))) AND Humans[Mesh])) NOT (((((((Infant[MeSH Terms]) OR Child[MeSH Terms]) OR Adolescent[MeSH Terms])) OR pregnancy) OR sleep apnea) AND Humans[Mesh])) AND Humans[Mesh])) NOT (Review[Publication Type] AND Humans[Mesh])) AND Humans[Mesh] AND English[lang])) NOT Case Reports[Publication Type]) AND Humans[Mesh] AND English[lang])) AND Humans[Mesh] AND English[lang])) NOT Qualitative Research[MeSH Terms]) AND Humans[Mesh] AND English[lang])

- Search Date: 2.7.18; 36 results

Psych Info

(Any Field: Index Terms: Sleep OR title: Sleep OR abstract: Sleep) AND (Any Field: Index Terms: Loneliness OR Any Field: Index Terms: Social Isolation OR title: Loneliness OR abstract: Loneliness OR title: Isolation OR abstract: Isolation OR title: Network size OR abstract: Network size OR title: Social participation OR abstract: Social Participation) NOT Index Terms: animal research NOT Index Terms: sleep apnea NOT Index Terms: pregnancy AND NOT Age Group: Childhood (birth-12 yrs) NOT Age Group: Adolescence (13-17 yrs) NOT Population Group: Animal NOT Methodology: Qualitative Study NOT Methodology: Clinical Case Study NOT Methodology: Literature Review AND Peer-Reviewed Journals only

- Search Date 2.2.18; 288 results

((title:(Insomnia) OR abstract:(Insomnia)) AND (AnyField:(Index Terms: Loneliness) OR AnyField:(Index Terms: Social Isolation) OR title:(Loneliness) OR abstract:(Loneliness) OR

title:(Isolation) OR abstract:(Isolation) OR title:(Network size) OR abstract:(Network size) OR title:(Social participation) OR abstract:(Social Participation)) AND -(IndexTerms:(animal research)) AND -(IndexTerms:(sleep apnea)) AND -(IndexTerms:(pregnancy)) AND AND ((-AgeGroupFilt:("Childhood (birth-12 yrs)"))) AND ((-AgeGroupFilt:("Adolescence (13-17 yrs)"))) AND ((-PopulationGroupFilt:("Animal"))) AND ((-MethodologyFilt:("Qualitative Study"))) AND ((-MethodologyFilt:("Clinical Case Study"))) AND ((-MethodologyFilt:("Literature Review"))) AND ((-MethodologyFilt:("Literature Review")))) *NOT* ((AnyField:(Index Terms: Sleep) OR title:(Sleep) OR abstract:(Sleep)) AND (AnyField:(Index Terms: Loneliness) OR AnyField:(Index Terms: Social Isolation) OR title:(Loneliness) OR abstract:(Loneliness) OR title:(Isolation) OR abstract:(Isolation) OR title:(Network size) OR abstract:(Network size) OR title:(Social participation) OR abstract:(Social Participation)) AND -(IndexTerms:(animal research)) AND -(IndexTerms:(sleep apnea)) AND -(IndexTerms:(pregnancy)) AND AND ((-AgeGroupFilt:("Childhood (birth-12 yrs)"))) AND ((-AgeGroupFilt:("Adolescence (13-17 yrs)"))) AND ((-PopulationGroupFilt:("Animal"))) AND ((-MethodologyFilt:("Qualitative Study"))) AND ((-MethodologyFilt:("Clinical Case Study"))) AND ((-MethodologyFilt:("Literature Review"))) AND ((-MethodologyFilt:("Literature Review")))) *AND* Peer-Reviewed Journals only

- Search date: 2.7.17, 33 results

((AnyField:(Index Terms: Sleep) OR title:(Sleep) OR abstract:(Sleep)) AND (title:("Living Alone") OR abstract:("Living Alone")) AND -(IndexTerms:(animal research)) AND -(IndexTerms:(sleep apnea)) AND -(IndexTerms:(pregnancy)) AND AND ((-AgeGroupFilt:("Childhood (birth-12 yrs)"))) AND ((-AgeGroupFilt:("Adolescence (13-17 yrs)"))) AND ((-PopulationGroupFilt:("Animal"))) AND ((-MethodologyFilt:("Qualitative

Study"))) AND ((-MethodologyFilt:("Clinical Case Study"))) AND ((-MethodologyFilt:("Literature Review"))) AND ((-MethodologyFilt:("Literature Review")))) *NOT* ((AnyField:(Index Terms: Sleep) OR title:(Sleep) OR abstract:(Sleep)) AND (AnyField:(Index Terms: Loneliness) OR AnyField:(Index Terms: Social Isolation) OR title:(Loneliness) OR abstract:(Loneliness) OR title:(Isolation) OR abstract:(Isolation) OR title:(Network size) OR abstract:(Network size) OR title:(Social participation) OR abstract:(Social Participation)) AND -(IndexTerms:(animal research)) AND -(IndexTerms:(sleep apnea)) AND -(IndexTerms:(pregnancy)) AND AND ((-AgeGroupFilt:("Childhood (birth-12 yrs)"))) AND ((-AgeGroupFilt:("Adolescence (13-17 yrs)"))) AND ((-PopulationGroupFilt:("Animal"))) AND ((-MethodologyFilt:("Qualitative Study"))) AND ((-MethodologyFilt:("Clinical Case Study"))) AND ((-MethodologyFilt:("Literature Review"))) AND ((-MethodologyFilt:("Literature Review")))) *AND* Peer-Reviewed Journals only

- Search date: 2.7.18, 26 results
