# Supplementary material for: Loneliness and sleep: A systematic review and meta-analysis
Source: Health Psychol Open. 2020 Apr 4;7(1):2055102920913235. doi: 10.1177/2055102920913235 (PMC7139193; doi:10.1177/2055102920913235)
Supplement: Supplementary_-_Appendix_B.__Ethnicity_for_studies_conducted_in_the_U.S. – Supplemental material for Loneliness and sleep: A systematic review and meta-analysis [file Supplementary_-_Appendix_B.__Ethnicity_for_studies_conducted_in_the_U.S..pdf]

Appendix B

Ethnicity for Studies Conducted in the U.S.

Author (Year)	%White	%Black	%Hispanic	%Asian/Pacific Islander	%Native American/Alaskan Native	%Other
Cacioppo, Hawkley, Bernston (2002)	Not reported					
Cacioppo, Hawkley, Crawford (2002) – Studies 1 & 2	Not reported					
Davis (2000)	10	64	26	0	0	0
Hawkley (2010)	35.8	35.4	28.8	0	0	0
Hays (1987)	54.6	15.7	14.7	10	0	5
Hom, Chu (2017) - Study 1	67.9	15.2	0	1.8	1.7	13.4
Hom, Chu (2017) - Study 2	66.2	14.8	13.5	4.3	1.1	
Hom, Chu (2017) - Study 3[a]	86.4	1.7	0	0	0.2	9.5
Hom, Hames (2017) - Study 1	75	21	<1	<1	<1	2
Hom, Hames (2017) - Study 2	65.2	15	13.5	4.3	1.1	0.9

Hom, Hames (2017) - Study 3	79.8	10.1	0	4.8	1	4.3
Hom, Hames (2017) - Study 4[b]	75.6	9.1	1.2	2.1	0.3	1.8
Hom, Hames (2017) - Study 5	61	24.8	10.5	1.2	1.2	1.2
Hom, Hames (2017) - Study 6	68	5	8	19	0	0
Jaremka (2014) - Study 1	85	Not reported				
Jaremka (2014) - Study 2	86	Not reported				
Kurina (2011)	100	0	0	0	0	0
Segrin (2010)	78	4	12	4	1	1
Segrin (2011)	77	1	12	6	1	2
Segrin (2015)	81.5	2	11	3	1	1.5
Zawadzki (2013) – Studies 3 & 4	Not reported					

Note: Superscripts denote the following: [a] Percentages add up to 97.8%. [b] Percentages add up to 90.1%
