# Supplementary material for: Loneliness and sleep: A systematic review and meta-analysis
Source: Health Psychol Open. 2020 Apr 4;7(1):2055102920913235. doi: 10.1177/2055102920913235 (PMC7139193; doi:10.1177/2055102920913235)
Supplement: Supplementary_-_Appendix_C._Measures – Supplemental material for Loneliness and sleep: A systematic review and meta-analysis [file Supplementary_-_Appendix_C._Measures.pdf]

Appendix C

Measures

Author (Year)	Sleep measure	Sleep Construct	Note on quality	Loneliness Measure	Direct /Indirect	Note on quality
Aanes (2011)	"How is your sleep in general?" on a 5-item Likert scale	Quality	Single item; Not assessed for psychometric properties	Six-item scale modified from scale developed for population-based research in Western Norway	Both	Modified scale; Acceptable internal consistency in this study (alpha=.77)
Cacioppo, Hawkley, Bernston (2002) - sleep efficiency	Blinking and movement during sleep via a Nightcap model P200B1	Both	Objective; Assessed for psychometric properties	UCLA Loneliness Scale (Revised)	Indirect	Scale; Assessed for psychometric properties

Author (Year)	Sleep measure	Sleep Construct	Note on quality	Loneliness Measure	Direct /Indirect	Note on quality
Cacioppo, Hawkley, Crawford, et al., (2002) - Study 1	Pittsburgh Sleep Quality Index (PSQI)	Quality	Scale; Assessed for psychometric properties	UCLA Loneliness Scale (Revised)	Indirect	Scale; Assessed for psychometric properties
Cacioppo, Hawkley, Crawford (2002) - Study 2	PSQI	Quality	Scale; Assessed for psychometric properties	UCLA Loneliness Scale (Revised)	Indirect	Scale; Assessed for psychometric properties
Cheng (2015)	PSQI (Chinese version)	Quality	Scale; Assessed for psychometric properties (in English and Chinese)	UCLA Loneliness Scale (Revised; Chinese Version)	Indirect	Scale; English version has been assessed for psychometric properties; Unclear if Chinese version has been assessed for psychometric properties but

Author (Year)	Sleep measure	Sleep Construct	Note on quality	Loneliness Measure	Direct /Indirect	Note on quality
						does demonstrate excellent internal consistency (alpha=0.94)
Christiansen (2016)	Single Item ratings for (1) general sleep quality on a 4-item Likert scale and (2) duration (how many hours and minutes do you approximately sleep on a weekday)	Both	Single item; Not assessed for psychometric properties	Hughes Loneliness Scale (Danish Version)	Indirect	Abbreviated (3-item) version of the UCLA Loneliness Scale English version has been assessed for psychometric properties; Acceptable internal consistency in this study (.72)
Chu (2016)	Insomnia Severity Index (ISI) translated into Korean	Insomnia	Scale; Assessed for psychometric properties in English, though it is unclear how translation	Thwarted Belonginess Subscale of the Interpersonal	Indirect	Scale; English version has been assessed for psychometric properties; Unclear if Korean version has

Author (Year)	Sleep measure	Sleep Construct	Note on quality	Loneliness Measure	Direct /Indirect	Note on quality
			might affect these properties	Needs Questionnaire (Korean Version)		been assessed for psychometric properties
Davis (2000)	7 questions on sleep from a larger questionnaire	Quality (but both measured)	Minimal information on the scale provided in the article. The cited article on the scale did not give any information on the scale or its psychometric properties.	Part of larger questionnaire; Appears to be a single-item on loneliness but not clearly specified	Not specified, but likely direct	Minimal information on the scale provided in the article; Appears to be a single question that has not been assessed for psychometric properties
Hawkley (2010)	Sleep Diary: Self-reported sleep duration and time in bed awake	Both	Administered daily, thus reducing retrospective reporting bias; Psychometric properties not reported	Six questions form the UCLA Loneliness Scale + one additional	Both	Modified scale; UCLA Loneliness Scale assessed for psychometric properties but the modified scale has not

Author (Year)	Sleep measure	Sleep Construct	Note on quality	Loneliness Measure	Direct /Indirect	Note on quality
				item (I feel lonely)		been assessed for psychometric properties
Hayley (2017)	Single item from the depression subscale of the Hopkins Symptoms Checklist asking about difficulty initiating and maintaining sleep	Quality	Single item; Not assessed for psychometric properties	Social and Emotional Loneliness Scale	Indirect	Poor face validity: appears to be tapping into social support and isolation rather than loneliness; Scale; Assessed for psychometric properties
Hays (1987)	Hours of sleep obtained on the average night	Quantity	Single item; Not assessed for psychometric properties	Three versions of the UCLA Loneliness Scale (ULS-20, ULS-8, ULS-4)	Indirect	assessed for psychometric properties; indirect

Author (Year)	Sleep measure	Sleep Construct	Note on quality	Loneliness Measure	Direct /Indirect	Note on quality
Hom, Chu (2017) - Study 1	5-item version of the ISI	Insomnia	Abbreviated scale; Full ISI has been assessed for psychometric properties; Abbreviated scale demonstrated good internal consistency in this study (alpha=.82)	5 questions from the Thwarted Belongingness Subscale of the Interpersonal Needs Questionnaire	Indirect	Abbreviated scale; Full scale assessed for psychometric properties; Abbreviated scale demonstrated good internal consistency in this study (alpha=.89)
Hom, Chu (2017) - Study 2	5-item version of the ISI	Insomnia	Abbreviated scale; Full ISI has been assessed for psychometric properties; Abbreviated scale demonstrated good internal consistency in this study (alpha=.87)	4 questions from the Thwarted Belongingness Subscale of the Interpersonal Needs Questionnaire	Indirect	Abbreviated scale; Full scale assessed for psychometric properties; Abbreviated scale demonstrated excellent internal consistency in this study (alpha=.91)

Author (Year)	Sleep measure	Sleep Construct	Note on quality	Loneliness Measure	Direct /Indirect	Note on quality
Hom, Chu (2017) - Study 3	ISI	Insomnia	Scale; Assessed for psychometric properties	Thwarted Belonginess Subscale of the Interpersonal Needs Questionnaire	Indirect	Scale; Assessed for psychometric properties
Hom, Hames (2017) - Study 1	ISI	Insomnia	Scale; Assessed for psychometric properties	Study-specific loneliness index created with items from CESD, PTSD checklist, Interpersonal Needs Questionnaire, and State Loss	Both	Scale; Not assessed for psychometric properties; Demonstrated good internal consistency in this study (alpha=0.85)

Author (Year)	Sleep measure	Sleep Construct	Note on quality	Loneliness Measure	Direct /Indirect	Note on quality
				of Interest and Pleasure Scale		
Hom, Hames (2017) - Study 2	5-item version of the ISI	Insomnia	Abbreviated scale; Full ISI has been assessed for psychometric properties; Abbreviated scale demonstrated good internal consistency in this study (alpha=.87)	4 questions from the Thwarted Belongingness Subscale of the Interpersonal Needs Questionnaire	Indirect	Abbreviated scale; Full scale assessed for psychometric properties; Abbreviated scale demonstrated excellent internal consistency in this study (alpha=.90)
Hom, Hames (2017) - Study 3	ISI	Insomnia	Scale; Assessed for psychometric properties	Thwarted Belonginess Subscale of the Interpersonal Needs Questionnaire	Indirect	Scale; Assessed for psychometric properties

Author (Year)	Sleep measure	Sleep Construct	Note on quality	Loneliness Measure	Direct /Indirect	Note on quality
Hom, Hames (2017) - Study 4	ISI	Insomnia	Scale; Assessed for psychometric properties	Thwarted Belonginess Subscale of the Interpersonal Needs Questionnaire	Indirect	Scale; Assessed for psychometric properties
Hom, Hames (2017) - Study 5	Single item from the BDI-II	Change in sleep	Single item; Not assessed for psychometric properties; Does not indicate direction of change (sleeping more or less)	9 questions from the Suicide Probability Scale used to form a loneliness subscale	Both	Scale; Not assessed for psychometric properties; Scale demonstrated adequate to good internal consistency in this study across time points (alpha=0.69-0.83)
Hom, Hames (2017) - Study 6	4-items from the Sleep Hopelessness Depression	Insomnia	Modified scale; Demonstrated adequate to good internal	UCLA Loneliness Scale (Revised)	Indirect	Scale; Assessed for psychometric properties

Author (Year)	Sleep measure	Sleep Construct	Note on quality	Loneliness Measure	Direct /Indirect	Note on quality
	Symptom Questionnaire		consistency (alpha=.79-.87)			
Jacobs (2006)	Yes/No question "Are you satisfied with your sleep in the last month?"	Sleep satisfaction	Single item; Not assessed for psychometric properties	Not specified in article but likely an item from the Brief Symptoms Inventory	Not specified, but likely direct	Minimal information on the scale provided in the article; Appears to be a single question that has not been assessed for psychometric properties
Jaremka (2014) - Study 1	PSQI	Quality	Scale; Assessed for psychometric properties	New York University (NYU) Loneliness Scale	Direct	Scale; Assessed for psychometric properties
Jaremka (2014) - Study 2	Not clearly specified but appears to be a single question asking participants to	Adequacy	Single item; Not assessed for psychometric properties	3 questions from the NYU Loneliness Scale	Direct	Abbreviated scale; Full scale assessed for psychometric properties; No evidence that

Author (Year)	Sleep measure	Sleep Construct	Note on quality	Loneliness Measure	Direct /Indirect	Note on quality
	compare the amount of sleep over the past three days to optimal amount					subscale has been assessed for psychometric properties
Kurina (2011)	(1) Wrist actigraphy: sleep duration and sleep fragmentation (2) PSQI	Both	(1) Objective; Assessed for psychometric properties (convergent validity with polysomnography for sleep duration and sleep fragmentation) (2) Scale; Assessed for psychometric properties	Hughes Loneliness Scale	Indirect	Abbreviated (3-item) version of the UCLA Loneliness Scale; Assessed for psychometric properties
Matthews (2017)	PSQI	Quality	Scale; Assessed for psychometric properties	4 questions from the UCLA	Indirect	Abbreviated scale; Full scale assessed for psychometric properties and abbreviated

Author (Year)	Sleep measure	Sleep Construct	Note on quality	Loneliness Measure	Direct /Indirect	Note on quality
				Loneliness Scale (Version 3)		scale includes all three Hughes Loneliness Scale Items; Abbreviated scale demonstrated good internal consistency in this study (alpha=0.83)
McHugh (2011)	PSQI	Quality	Scale; Assessed for psychometric properties	De Jong Gierveld Loneliness Scale (6-Item)	Indirect	Scale; Assessed for psychometric properties
McHugh (2013)	PSQI	Quality	Scale; Assessed for psychometric properties	De Jong Gierveld Loneliness Scale (6-Item)	Indirect	Scale; Assessed for psychometric properties
O'Connell (2016)	4 items on sleep disturbance taken	Quality	Abbreviated scale; Full health scale has been	National Institute of	Both	Scale; Assessed for psychometric properties

Author (Year)	Sleep measure	Sleep Construct	Note on quality	Loneliness Measure	Direct /Indirect	Note on quality
	from the 14-item Physical Health Questionnaire		assessed for psychometric properties but the properties of the abbreviated scale are not clear	Health Toolbox of Adult Social Relationships Loneliness Scale		
Segrin (2010)	Sleep subscale from the Health Practices Scale	Adequacy	Subscale; Full Health Practices Scale only assessed for internal consistency in cited article (Jackson, 2006); Subscale demonstrated good internal consistency in this study (alpha=.83)	UCLA Loneliness Scale (Version 3)	Indirect	Scale; Assessed for psychometric properties

Author (Year)	Sleep measure	Sleep Construct	Note on quality	Loneliness Measure	Direct /Indirect	Note on quality
Segrin (2011)	PSQI	Quality	Scale; Assessed for psychometric properties	UCLA Loneliness Scale (Version 3)	Indirect	Scale; Assessed for psychometric properties
Segrin (2015)	PSQI	Quality	Scale; Assessed for psychometric properties	UCLA Loneliness Scale (Version 3)	Indirect	Scale; Assessed for psychometric properties
Smith (2010)	PSQI	Quality	Scale; Assessed for psychometric properties	UCLA Loneliness Scale (Revised)	Indirect	Scale; Assessed for psychometric properties
Steptoe (2004)	Jenkins et al. (1988) Scale	Quality	Scale; Assessed for psychometric properties	UCLA Loneliness Scale (Revised)	Indirect	Scale; Assessed for psychometric properties
Stickley (2015)	Not clearly specified but appears to be single yes/no question on	Insomnia	Single item; Not assessed for psychometric properties	"How often do you feel lonely?" on a 4-item Likert scale	Direct	Single item; Not assessed for psychometric properties

Author (Year)	Sleep measure	Sleep Construct	Note on quality	Loneliness Measure	Direct /Indirect	Note on quality
	experience of insomnia in the past year					
Yu (2017)	(1 – in 2000) Sleep item from the Center for Epidemiologic Studies Depression Scale (CES-D)<br><br>(2 – in 2006) PSQI (Chinese version)	Quality	(1) Single item; Not assessed for psychometric properties<br><br>(2) Scale; Assessed for psychometric properties (in English and Chinese)	Loneliness item from the CES-D	Direct	Single item; Not assessed for psychometric properties
Zawadzki (2013) - Study 3	PSQI	Quality	Scale; Assessed for psychometric properties	UCLA Loneliness Scale (Version 3)	Indirect	Scale; Assessed for psychometric properties

Author (Year)	Sleep measure	Sleep Construct	Note on quality	Loneliness Measure	Direct /Indirect	Note on quality
Zawadzki (2013) - Study 4	PSQI	Quality	Scale; Assessed for psychometric properties	UCLA Loneliness Scale (Version 3)	Indirect	Scale; Assessed for psychometric properties

Note. The leading author(s) and year is underlined for longitudinal research.
