# Supplementary material for: Loneliness and sleep: A systematic review and meta-analysis
Source: Health Psychol Open. 2020 Apr 4;7(1):2055102920913235. doi: 10.1177/2055102920913235 (PMC7139193; doi:10.1177/2055102920913235)
Supplement: Supplementary_-_Appendix_D._Psychometric_properties_of_scales – Supplemental material for Loneliness and sleep: A systematic review and meta-analysis [file Supplementary_-_Appendix_D._Psychometric_properties_of_scales.pdf]

Appendix D

Psychometric properties of scales

Sleep

Health Practices Scale.

- Internal consistency: α=.89 for sleep adequacy
- No other assessment of psychometric properties in Jackson, 2006.

Insomnia Severity Index (ISI).

- Internal consistency: α=.74-.79
- Concurrent validity: correlates with total score on relevant polysomnography
- Convergent validity: correlates with measrues of fatigue, quality of life, anxiety, and depression (Smith & Wegener, 2003)

Jenkins Scale for the Estimation of Sleep Problems in Clinical Research.

- Internal consistency: α=.63-.79
- Convergent validity: correlates with measure of fatigue and use of certain medications (Jenkins, Stanton, Niemcryk, & Rose, 1988)
- Test-retest reliability: r=.42 (one-year, unstable sample)

Pittsburgh Sleep Quality Index (PSQI).

- Internal consistency: α=.83

- Test-retest reliability: r=.65-.84 depending on sleep component (one year)
- Criterion validity: PSQI distinguishes between good and poor sleepers according to structured clinical interviews, physical exam, and polysomnographic testing
- Convergent validity: correlates with daily sleep diaries (PSQI: longer sleep latency and shorter sleep duration), correlates with other sleep measures
- Divergent validity: poorly correlates with measures examining nausea, vomiting, and taste changes (Smith & Wegener, 2003)

Loneliness

The Hughes Loneliness Scale.

- Internal consistency: α =.72
- Discriminant validity: correlates with depression and perceived stress, but weaker correlations with other emotions such as enjoyment, energy, motivation (Hughes, Waite, Hawkley, & Cacioppo, 2004)

The Jong Gierveld Scale of Loneliness (6-item).

- Face validity: Some items appear to be tapping into different constructs, such as depression (e.g., “I experience a general sense of emptiness”) and social support (“There are plenty of people I can rely on when I have problems”)
- Internal consistency: α =.70-.76
- Convergent validity: correlates with partner status, subjective health, and other versions of the Jong Gierveld Scale of Loneliness (Gierveld & Tilburg, 2006)

New York University (NYU) Loneliness Scale.

- Internal consistency: α = .88-.89
- Convergent validity: correlates with parental divorce before age 18, quality of relationships with parents, depression, self-esteem, psychosomatic symptoms (Rubenstein & Shaver, 1982)

The Social and Emotional Loneliness Scale.

- Face validity: Some items appear to be tapping into different constructs, such as social support (e.g. "I have a romantic partner who gives me support and encouragement") and network (e.g., "I belong to a network of friends", "I don't have a special love relationship").
- Internal consistency: α =.76-.78 (Green, Richardson, Lago, & Shatton-Jones, 2001)
- Convergent validity: correlates with other loneliness scales (Hayley et al., 2017)

Thwarted Belongingness Subscale of the Interpersonal Needs Questionnaire.

- Internal consistency: α =.85 (Van Orden, Witte, Gordon, Bender, & Joiner, 2008)
- Concurrent validity: Correlates with higher severity of suicidal ideation
- Predictive validity: thwarted belongingness predicts odds of suicidal ideation 1 month later
- Convergent validity: correlates with other scales of loneliness, social support (Van Orden, Cukrowicz, Witte, & Joiner, 2012)

The UCLA Loneliness Scale.

- Internal consistency: α=.96

- Test-retest reliability: r=.73 (2 months)
- Convergent validity: correlates with self-reports of current loneliness, related emotional states, and volunteering for loneliness clinic (Russell, Peplau, & Ferguson, 1978)

The Revised UCLA Loneliness Scale.

- Revises UCLA loneliness scale to include reverse coding (reducing risk of response bias)
- Internal consistency: α=.94.
- Convergent validity: correlates with other measures of loneliness, negative affect, social risk taking, and affective tendencies
- Discriminant validity: low correlation with measure of social desirability (Russell, Peplau, & Cutrona, 1980)

The UCLA Loneliness Version 3.

- Simplified Revised UCLA Loneliness Scale to increase readability (e.g., eliminated a double negative, use of the word "superficial")
- Internal consistency: α =.89-.94
- Test-retest reliability: r = .73 (one year)
- Convergent validity: correlates with other measures of loneliness, the adequacy of interpersonal relationships, health and well-being (Russell, 1996)
