# Supplementary material for: Loneliness and sleep: A systematic review and meta-analysis
Source: Health Psychol Open. 2020 Apr 4;7(1):2055102920913235. doi: 10.1177/2055102920913235 (PMC7139193; doi:10.1177/2055102920913235)
Supplement: Supplementary_-_Appendix_E._Summary_of_meta-analyses – Supplemental material for Loneliness and sleep: A systematic review and meta-analysis [file Supplementary_-_Appendix_E._Summary_of_meta-analyses.pdf]

Appendix E

Summary of meta-analyses

	k	n	Weighted mean effect size (*r*)	CI Lower	CI Upper	*Q*-Statistic
Outlier excluded						
All studies with information available to calculate *r* included						
Sleep disturbance	24	34,254	.27	.24	.30	90.66
Insomnia symptoms	9	10,236	.28	.24	.33	25.11
Sleep quality	15	24,018	.26	.22	.31	65.23
Outlier excluded						
Studies with effect size estimates that violate the normality distribution assumptions excluded						
Sleep disturbance	18	20,218	.28	.24	.31	60.25
Insomnia symptoms	8	9,046	.29	.24	.34	24.96
Sleep quality	10	11,172	.27	.22	.32	32.39
Outlier included						
All studies with information available to calculate *r* included						
Sleep disturbance	25	34,671	.29	.25	.34	161.42

Insomnia symptoms	10	10,653	.33	.24	.40	92.40
Outlier included						
Studies with effect size estimates that violate the normality distribution assumptions excluded						
Sleep disturbance	19	20,635	.30	.24	.35	124.04
Insomnia symptoms	9	9,463	.33	.24	.42	91.47

Notes. (1) Sleep disturbance is defined as self-reported sleep quality and insomnia symptoms. (2) Analyses only evaluated self-reported sleep quality; see Figure 3 for other effect sizes. (3) CI = Confidence Interval.
