# Supplementary material for: Loneliness and sleep: A systematic review and meta-analysis
Source: Health Psychol Open. 2020 Apr 4;7(1):2055102920913235. doi: 10.1177/2055102920913235 (PMC7139193; doi:10.1177/2055102920913235)
Supplement: Supplementary_-_Appendix_F._Baujat – Supplemental material for Loneliness and sleep: A systematic review and meta-analysis [file Supplementary_-_Appendix_F._Baujat.pdf]

Appendix F

Baujat Plot

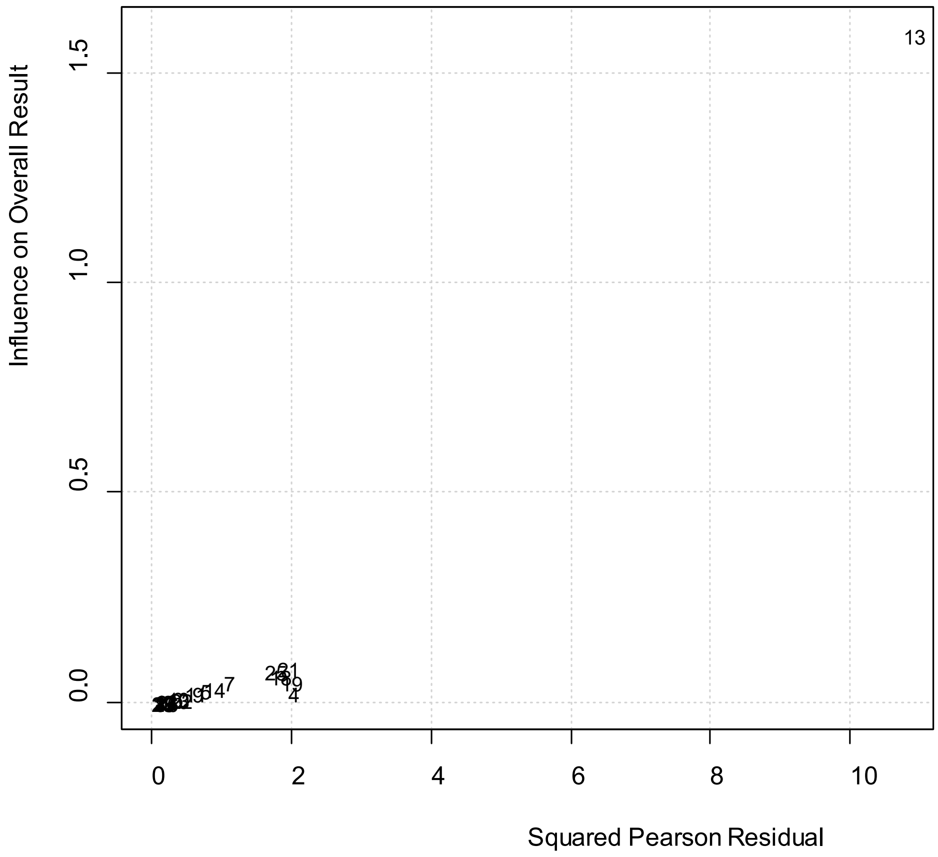

Note. Study 13 (top right corner) is the third study presented in the manuscript by Hom, Chu et al. (2017).
