# Supplementary figures and images for: Loneliness and sleep: A systematic review and meta-analysis
Source: Health Psychol Open. 2020 Apr 4;7(1):2055102920913235. doi: 10.1177/2055102920913235 (PMC7139193; doi:10.1177/2055102920913235)

Appendix G  
Funnel Plot

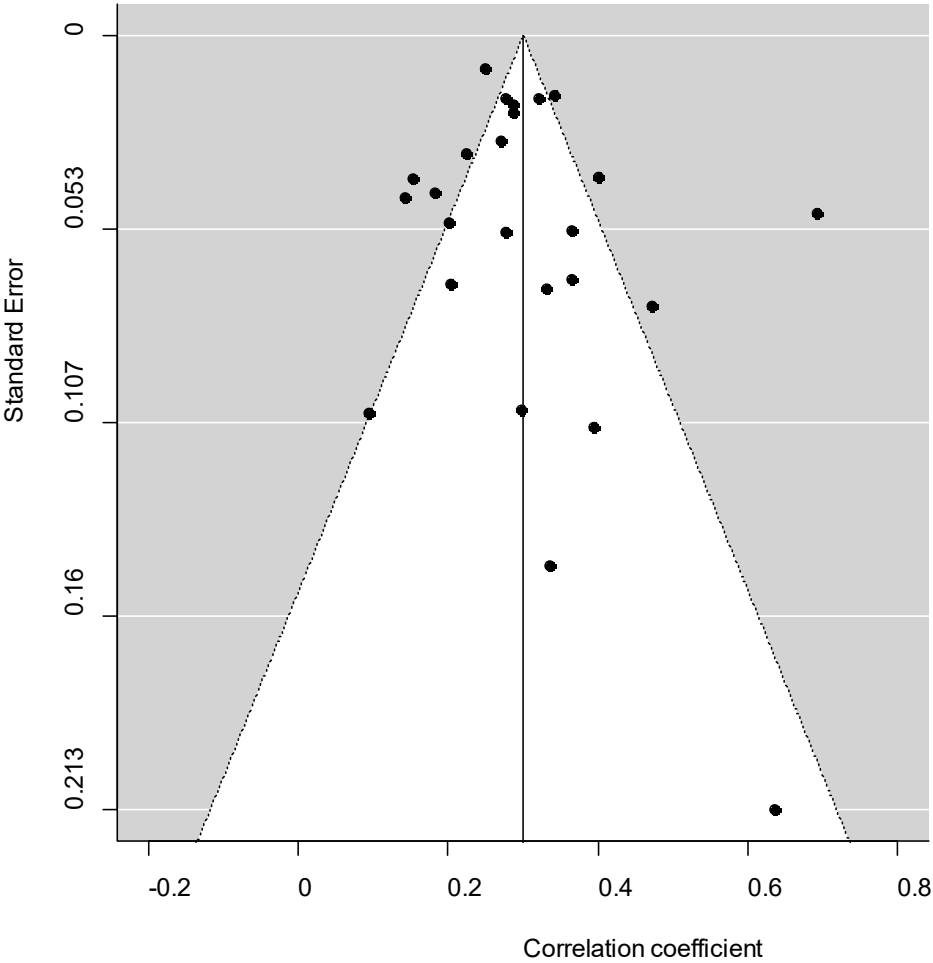

Supplement: Supplementary_-_Appendix_G._Funnel_Plot – Supplemental material for Loneliness and sleep: A systematic review and meta-analysis [file Supplementary_-_Appendix_G._Funnel_Plot.pdf]
