# Supplementary material for: Loneliness and sleep: A systematic review and meta-analysis
Source: Health Psychol Open. 2020 Apr 4;7(1):2055102920913235. doi: 10.1177/2055102920913235 (PMC7139193; doi:10.1177/2055102920913235)
Supplement: Supplementary_-_Appendix_H._Citations_for_Articles_Identified_in_Systematic_Review – Supplemental material for Loneliness and sleep: A systematic review and meta-analysis [file Supplementary_-_Appendix_H._Citations_for_Articles_Identified_in_Systematic_Review.pdf]

Appendix H

Citations for Articles Identified in Systematic Review

Aanes, M. M., Hetland, J., Pallesen, S., & Mittelmark, M. B. (2011). Does loneliness mediate the stress-sleep quality relation? the Hordaland Health Study. *International Psychogeriatrics*, *23*(6), 994–1002. https://doi.org/10.1017/S1041610211000111

Cacioppo, J. T., Hawkley, L. C., Bernston, G. C., Ernst, J. M., Gibbs, A. C., Stickgold, R., & Hobson, J. A. (2002). Do lonely days invade the nights? Potential social modualation of sleep efficiency. *Psychological Science*, *13*(4), 384–387. https://doi.org/10.1111/j.0956-7976.2002.00469.x

Cacioppo, J. T., Hawkley, L. C., Crawford, E., Ernst, J. M., Burleson, M. H., Kowalewski, R. B., … Berntson, G. G. (2002). Loneliness and health: Potential mechanisms. *Psychosomatic Medicine*, *64*(3), 407–417.

Cheng, P., Jin, Y., Sun, H., Tang, Z., Zhang, C., Chen, Y., … Huang, F. (2015). Disparities in prevalence and risk indicators of loneliness between rural empty nest and non-empty nest older adults in Chizhou, China. *Geriatrics and Gerontology International*, *15*(3), 356–364. https://doi.org/10.1111/ggi.12277

Christiansen, J., Larsen, F. B., & Lasgaard, M. (2016). Do stress, health behavior, and sleep mediate the association between loneliness and adverse health conditions among older people? *Social Science & Medicine*, *152*, 80–86. https://doi.org/10.1016/j.socscimed.2016.01.020

Chu, C., Hom, M. A., Rogers, M. L., Ringer, F. B., Hames, J. L., Suh, S., & Joiner, T. E. (2016). Is Insomnia Lonely ? Exploring Thwarted Belongingness as an Explanatory Link between Insomnia and Suicidal Ideation in a Sample of South Korean University Students, *12*(5),

647–652.

Davis, J. E., & Shuler, P. A. (2000). A biobehavioral framework for examining altered sleep-wake patterns in homeless women. *Issues in Mental Health Nursing*, *21*(2), 171–183. https://doi.org/10.1080/016128400248176

Hawkley, L. C., Preacher, K. J., & Cacioppo, J. T. (2010). Loneliness impairs daytime functioning but not sleep duration. *Health Psychology*, *29*(2), 124–129. https://doi.org/10.1037/a0018646

Hayley, A. C., Downey, L. A., Stough, C., Sivertsen, B., Knapstad, M., Øverland, S., … Ober, C. (2017). Social and emotional loneliness and self-reported difficulty initiating and maintaining sleep (DIMS) in a sample of Norwegian university students. *Scandinavian Journal of Psychology*, *58*(1), 91–99. https://doi.org/10.1111/sjop.12343

Hays, R. D., & DiMatteo, M. R. (1987). A short-form measure of loneliness. *Journal of Personality Assessment*, *51*(1), 69–81. https://doi.org/10.1207/s15327752jpa5101_6

Hom, M. A., Chu, C., Schneider, M. E., Lim, I. C., Hirsch, J. K., Gutierrez, P. M., & Joiner, T. E. (2017). Thwarted belongingness as an explanatory link between insomnia symptoms and suicidal ideation: Findings from three samples of military service members and veterans. *Journal of Affective Disorders*, *209*, 114–123. https://doi.org/10.1016/j.jad.2016.11.032

Hom, M. A., Hames, J. L., Bodell, L. P., Buchman-Schmitt, J. M., Chu, C., Rogers, M. L., … Joiner, T. E. (2017). Investigating insomnia as a cross-sectional and longitudinal predictor of loneliness: Findings from six samples. *Psychiatry Research*, *253*, 116–128. https://doi.org/10.1016/j.psychres.2017.03.046

Jacobs, J. M., Cohen, A., Hammerman-Rozenberg, R., & Stessman, J. (2006). Global sleep satisfaction of older people: the Jerusalem Cohort Study. *Journal of the American*

Geriatrics Society, *54*(2), 325–329. https://doi.org/10.1111/j.1532-5415.2005.00579.x

Jaremka, L. M., Andridge, R. R., Fagundes, C. P., Alfano, C. M., Povoski, S. P., Lipari, A. M., … Kiecolt-Glaser, J. K. (2014). Pain, depression, and fatigue: Loneliness as a longitudinal risk factor. *Health Psychology*, *33*(9), 948–957. https://doi.org/10.1037/a0034012

Kurina, L. M., Knutson, K. L., Hawkley, L. C., Cacioppo, J. T., Lauderdale, D. S., & Ober, C. (2011). Loneliness Is Associated with Sleep Fragmentation in a Communal Society. *Sleep*, *34*(11), 1519–1526. https://doi.org/10.5665/sleep.1390

Matthews, T., Danese, A., Gregory, A. M., Caspi, A., Moffitt, T. E., & Arseneault, L. (2017). Sleeping with one eye open: loneliness and sleep quality in young adults. *Psychological Medicine*, *47*(12), 2177–2186. https://doi.org/10.1017/S0033291717000629

McHugh, J. E., Casey, A. M., & Lawlor, B. A. (2011). Psychosocial correlates of aspects of sleep quality in community-dwelling Irish older adults. *Aging & Mental Health*, *15*(6), 749–755. https://doi.org/10.1080/13607863.2011.562180

McHugh, J. E., & Lawlor, B. A. (2013). Perceived stress mediates the relationship between emotional loneliness and sleep quality over time in older adults. *British Journal of Health Psychology*, *18*(3), 546–555. https://doi.org/10.1111/j.2044-8287.2012.02101.x

O'Connell, B. H., O'Shea, D., & Gallagher, S. (2016). Mediating effects of loneliness on the gratitude-health link. *Personality and Individual Differences*, *98*, 179–183. https://doi.org/10.1016/j.paid.2016.04.042

Segrin, C., & Burke, T. J. (2015). Loneliness and Sleep Quality: Dyadic Effects and Stress Effects. *Behavioral Sleep Medicine*, *13*(3), 241–254. https://doi.org/10.1080/15402002.2013.860897

Segrin, C., & Domschke, T. (2011). Social support, loneliness, recuperative processes, and their

direct and indirect effects on health. *Health Communication*, *26*(3), 221–232. https://doi.org/10.1080/10410236.2010.546771

Segrin, C., & Passalacqua, S. A. (2010). Functions of loneliness, social support, health behaviors, and stress in association with poor health. *Health Communication*, *25*(4), 312–322. https://doi.org/10.1080/10410231003773334

Smith, S. S., Kozak, N., & Sullivan, K. A. (2010). An investigation of the relationship between subjective sleep quality, loneliness and mood in an Australian sample: Can daily routine explain the links? *International Journal of Social Psychiatry*, *58*(2), 166–171. https://doi.org/10.1177/0020764010387551

Steptoe, A., Owen, N., Kunz-Ebrecht, S. R., & Brydon, L. (2004). Loneliness and neuroendocrine, cardiovascular, and inflammatory stress responses in middle-aged men and women. *Psychoneuroendocrinology*, *29*(5), 593–611. https://doi.org/10.1016/S0306-4530(03)00086-6

Stickley, A., Koyanagi, A., Leinsalu, M., Ferlander, S., Sabawoon, W., & McKee, M. (2015). Loneliness and health in Eastern Europe: Findings from Moscow, Russia. *Public Health*, *129*(4), 403–410. https://doi.org/10.1016/j.puhe.2014.12.021

Yu, B., Steptoe, A., Niu, K., Ku, P.-W., & Chen, L.-J. (2017). Prospective associations of social isolation and loneliness with poor sleep quality in older adults. *Quality of Life Research*, *27*(3), 683–691. https://doi.org/10.1007/s11136-017-1752-9

Zawadzki, M. J., Graham, J. E., & Gerin, W. (2013). Rumination and anxiety mediate the effect of loneliness on depressed mood and sleep quality in college students. *Health Psychology*, *32*(2), 212–222. https://doi.org/10.1037/a0029007
